# (*R*)-ketamine restores anterior insular cortex activity and cognitive deficits in social isolation-reared mice

**DOI:** 10.1038/s41380-024-02419-6

**Published:** 2024-02-23

**Authors:** Rei Yokoyama, Yukio Ago, Hisato Igarashi, Momoko Higuchi, Masato Tanuma, Yuto Shimazaki, Takafumi Kawai, Kaoru Seiriki, Misuzu Hayashida, Shun Yamaguchi, Hirokazu Tanaka, Takanobu Nakazawa, Yasushi Okamura, Kenji Hashimoto, Atsushi Kasai, Hitoshi Hashimoto

**Affiliations:** 1 Laboratory of Molecular Neuropharmacology, Graduate School of Pharmaceutical Sciences, Osaka University, Suita, Osaka 565-0871 Japan; 2 Department of Cellular and Molecular Pharmacology, Graduate School of Biomedical and Health Sciences, Hiroshima University, Hiroshima, Hiroshima 734-8553 Japan; 3 Laboratory of Integrative Physiology, Department of Physiology, Graduate School of Medicine, Osaka University, Suita, Osaka 565-0871 Japan; 4 Department of Morphological Neuroscience, Graduate School of Medicine, Gifu University, Gifu, Gifu 501-1194 Japan; 5 Center for One Medicine Innovative Translational Research, Institute for Advanced Study, Gifu University, Gifu, Gifu 501-1194 Japan; 6 Faculty of Information Technology, Tokyo City University, Setagaya, Tokyo 158-8557 Japan; 7 Department of Bioscience, Tokyo University of Agriculture, Setagaya, Tokyo 156-8502 Japan; 8 Graduate School of Frontier Biosciences, Osaka University, Suita, Osaka 565-0871 Japan; 9 Division of Clinical Neuroscience, Chiba University Center for Forensic Mental Health, Chuo, Chiba 260-8670 Japan; 10 Systems Brain Science Project, Drug Innovation Center, Graduate School of Pharmaceutical Sciences, Osaka University, Suita, Osaka 565-0871 Japan; 11 Molecular Research Center for Children’s Mental Development, United Graduate School of Child Development, Osaka University, Kanazawa University, Hamamatsu University School of Medicine, Chiba University, and University of Fukui, Suita, Osaka 565-0871 Japan; 12 Division of Bioscience, Institute for Datability Science, Osaka University, Suita, Osaka 565-0871 Japan; 13 Open and Transdisciplinary Research Initiatives, Osaka University, Suita, Osaka 565-0871 Japan; 14 Department of Molecular Pharmaceutical Sciences, Graduate School of Medicine, Osaka University, Suita, Osaka 565-0871 Japan

**Keywords:** Neuroscience, Cell biology

## Abstract

Chronic social isolation increases the risk of mental health problems, including cognitive impairments and depression. While subanesthetic ketamine is considered effective for cognitive impairments in patients with depression, the neural mechanisms underlying its effects are not well understood. Here we identified unique activation of the anterior insular cortex (aIC) as a characteristic feature in brain-wide regions of mice reared in social isolation and treated with (*R*)-ketamine, a ketamine enantiomer. Using fiber photometry recording on freely moving mice, we found that social isolation attenuates aIC neuronal activation upon social contact and that (*R*)-ketamine, but not (*S*)-ketamine, is able to counteracts this reduction. (*R*)-ketamine facilitated social cognition in social isolation-reared mice during the social memory test. aIC inactivation offset the effect of (*R*)-ketamine on social memory. Our results suggest that (*R*)-ketamine has promising potential as an effective intervention for social cognitive deficits by restoring aIC function.

## Introduction

Chronic social isolation stress has a negative impact on individual mental health, causing social cognitive dysfunctions and depression, regardless of whether it occurs during the juvenile or adult stages [1–4]. Particularly reduced social interactions during childhood have been found to significantly contribute to impairments in adult social cognition [5]. In rodent models, social isolation paradigms lasting >2 weeks have been shown to lead to emotional abnormalities, including reduced sociability [6–10]. The maintenance of social cognitive function involves the engagement of various brain regions, such as the insular and prefrontal cortices [11–13]. However, whether these brain regions are involved in the recovery of social cognitive dysfunction, particularly in response to therapeutic interventions, is unclear.

Ketamine, a racemic mixture of (*S*)- and (*R*)-ketamine, is recognized for its rapid antidepressant effects and amelioration of negative emotional behaviors [14–18], and its potential to improve cognitive dysfunctions in rodent models of depression and patients with depression [19–22]. It has been reported that the rapid antidepressant effects of ketamine are due to neuroplastic changes in the prefrontal cortex, brain-derived neurotrophic factor (BDNF)-tropomyosin receptor kinase B (TrkB) signaling activation, and suppression of neural bursts in the habenula [23–27]. However, the effectiveness of each ketamine enantiomer remains controversial, and understanding the underlying neural mechanisms is crucial for further development of ketamine-based therapies. Studies, including ours, have revealed differences in the molecular properties of the enantiomers [25, 28–30]. For example, (*S*)-ketamine has higher *N*-methyl-D-aspartate signal inhibitory potency and enhances neuronal activity in the ventromedial prefrontal cortex than (*R*)-ketamine [31–33]. Furthermore, (*R*)-ketamine induces serotonin release in the prefrontal cortex, whereas (*S*)-ketamine induces dopamine release, indicating differences in pharmacological effects between the two enantiomers [34]. However, the neural mechanisms underlying the differential pharmacological effects of these ketamine enantiomers remain unknown.

In this study, we compared the effects of ketamine enantiomers on behavioral and social cognitive dysfunction, brain-wide neuronal activation map and neuronal activity during social behavior in chronic social isolation-reared mice. We found that (*R*)-ketamine, but not (*S*)-ketamine, enhances the neuronal activity of the anterior insular cortex (aIC) and decreases social cognitive dysfunction induced by social isolation rearing.

## Materials and methods

### Animals

All animal studies were approved by the Animal Care and Use Committee of the Graduate School of Pharmaceutical Sciences, Osaka University (Douyaku R02-8). All efforts were made to minimize the number of animals used. The following adult male mice (9‒15 weeks of age) were used for behavioral tests and were randomly assigned numbers and tested blind to the experimental condition. Arc-dVenus reporter mice, which express the destabilized fluorescent protein dVenus driven by the *Arc* gene promoter on a C57BL/6 J background, were used for brain-wide mapping of immediate early genes (IEG) and a behavioral test; C57BL/6 J wild-type mice (SLC, Shizuoka, Japan) were also used for behavioral tests. For social isolation rearing, the mice were individually isolated starting at 3 weeks of age and kept in isolation for at least 6 weeks in opaque polypropylene cages (length × width × height: 24 × 17 × 12 cm), whereas the control group was housed under normal group-reared conditions (5 or 6 animals per cage) in same-sized clear plastic cages. They were kept on a 12 h light–dark cycle (lights on at 8:00 a.m.) with controlled room temperature and humidity, and water and food (CMF; Oriental Yeast, Osaka, Japan) available ad libitum.

### Ketamine administration

(*S*)- and (*R*)-ketamine hydrochloride were prepared as described previously [35]. In brief, (*R,S*)-ketamine (Ketalar; Daiichi Sankyo Pharmaceutical Ltd., Tokyo, Japan) was recrystallized with L-(+)-tartaric acid or D-(-)-tartaric acid to obtain (*S*)- and (*R*)-ketamine, respectively. Both were dissolved in saline (0.9% [w/v] solution of NaCl; Otsuka Pharmaceutical Ltd., Tokyo, Japan) just before use.

### Behavioral tests

#### Forced swim test (FST)

The Forced swim test was performed as previously reported [36]. In brief, swimming sessions were conducted by placing a mouse in an individual acrylic cylinder (25 cm height × 19 cm diameter) containing 25 ± 1 °C water at a depth of 13 cm so that the mouse could not support themselves by touching the bottom with their paws. The performance of the mouse for 6 min in the swimming session was videotaped for subsequent analysis. After the session, the mouse was removed from the cylinders, dried with paper towels and placed under a warming lamp until completely dry, and then, the mouse was returned to its home cage. The total duration of immobility during the 6 min period was measured by an observer blind to the treatment.

#### Three-chamber test

The three-chamber test was performed as previously reported [37]. The test mouse was placed in the central chamber of an opaque polyvinyl chloride box (length × width × height: 42 × 50 × 30 cm) divided into three interconnected chambers under 400 lx illumination (measured in the center zone). Clear partitions with openings allowed the mouse to move freely between the chambers. After a 90 min habituation period, an unfamiliar male mouse of the same strain but 1 week younger, along with an unfamiliar object, was introduced into separate wire-mesh intruder boxes (length × width × height: 10 × 6.5 × 20 cm). The test mouse explored both the intruder mouse and object for 10 min. “Mouse interaction” and “object interaction” were defined as the test mouse’s nose contact with each intruder box.

#### Five-trial social memory test

The five-trial social memory test was performed as previously reported [38]. The test mouse was habituated to a small cylindrical cage with bars placed in the home-cage under 400 lx illumination. After a 30 min habituation period, an unfamiliar male mouse of the same strain but 1 week younger was introduced into the cylindrical cage for four successive trials of 5 min each (10 min inter-trial interval). In the fifth trial, an unfamiliar mouse was presented. The performance of the mouse in the investigation session was videotaped. After each session, the cylindrical cage was removed from the home-cage. The total investigation time was measured during the 5 min period in each session by an observer blinded to the treatment.

### Brain-wide IEG mapping and machine learning classification

For brain-wide IEG mapping, we perfused mice and collected their brains 5 h after the FST. Whole-brain imaging at subcellular resolution was performed using the FAST system as described previously [39, 40]. The total number of dVenus-positive cells in each brain area in 22 semi-manually parcellated brain areas was determined and the spatial coordinates of all dVenus-positive cells in the IC were obtained using TRI/FCS-NUC64 (R.10.00.04.3-3-H-64; Ratoc System Engineering, Tokyo, Japan) [39, 41]. Using three group datasets (i.e., saline, (*R*)-ketamine, and (*S*)-ketamine; *n* = 5 per group), the total number of dVenus-positive cells in each area was standardized into z-score data. Linear support vector machine classifications for the datasets ((*R*)-ketamine vs. saline or (*S*)-ketamine) were implemented using the fitcsvm package in MATLAB R2022a. The classifier weights in each classification were transformed into corresponding activation patterns using the following formula [42]:

$$A={\Sigma }_{x}W{\Sigma }_{\hat{s}}^{-1},$$

where

$${\Sigma }_{\hat{S}}={W}^{T}{\Sigma }_{x}W$$

and *A* is the *M* × *K* activation pattern matrix in the forward model (*M*, the number of brain areas; *K*, the number of target variables in each area of the group); $x$ is the *M*-dimensional vector of the observed data (z-score in 22 areas, so *M* = 22 in our case); $\hat{s}$ is the *K*-dimensional vector of the latent factors; ${\Sigma }_{x}$ is a data covariance matrix; *W* is the *M* × *K* weight matrix in the backward model (classifier weights in a linear support vector machine classification); and ${\Sigma }_{\hat{S}}$ is a covariance matrix of the latent factors.

### Generation of AAV transgenes and virus production

For pAAV-CaMKIIα-hM4Di-mCherry, the hM4Di-mCherry cassette was cut from pAAV-hSyn-DIO-hM4Di-mCherry [41] using AscI (R0558, New England Biolabs, Ipswich, MA) and NheI-HF (R3131, New England Biolabs) and ligated with SalI-HF- and EcoRV-HF-digested pAAV-CaMKIIα-DIO-hM3Dq-mCherry [41] using a linker and the DNA Ligation Kit Mighty Mix (Takara Bio, Shiga, Japan).

pAAV-CaMKIIα-hM3Dq-mCherry and pAAV-CaMKIIα-mCherry were produced as previously described [41].

For pAAV-CaMKIIα-GCaMP6f-P2A-nls-dTomato, the GCaMP6f-P2A-nls-dTomato cassette was amplified from pAAV-EF1a-DIO-GCaMP6f-P2A-nls-dTomato (Addegene plasmid #51083; a gift from Jonathan Thing; RRID: Addgene_51083) using Q5 Hot Start High-Fidelity 2× Master Mix (New England Biolabs). Then, the amplified cassette was subcloned into the plasmid containing pAAV-CaMKIIα-mCherry using the In-Fusion® HD cloning kit (Clontech, Mountain View, CA) after excising the mCherry sequence.

For virus production and purification, AAV vectors were produced via the helper-free triple transfection procedure as described previously [41].

### Stereotaxic surgery

The mice were anesthetized using 2.0%–2.5% isoflurane (FUJIFILM Wako Pure Chemical Corp., Osaka, Japan) and placed in a stereotaxic apparatus (Narishige Scientific Instrument Lab., Tokyo, Japan). Microinjection into the aIC (stereotaxic coordinates: +2.1 mm anterior, ±2.6 mm lateral, and −3.7 mm ventral to the bregma) was performed using a Gastight syringe with a 33-gauge needle (Hamilton, Reno, NV) and a microinjection pump system UMP3T (World Precision Instruments, Sarasota, FL). The AAV mixture was then deposited at a rate of 100 nL/min. The needle was left in place for 5 min before withdrawal and then slowly removed to avoid backflow.

For photometry experiments, a TeleFipho fiber-optic cannula (fiber core at 400 μm/NA 0.39, cladding of 425 μm, ferrule diameter of 2.5 mm, cannula length of 3.5 mm; Nihon Bioresearch Inc., Aichi, Japan) was implanted over the right aIC (stereotaxic coordinates: +2.1 mm anterior, +2.6 mm lateral, and −3.5 mm ventral to the bregma) after AAV microinjection. The fiber-optic cannula was anchored to the skull using Super-Bond (Sun Medical Co. Ltd., Shiga, Japan) and UNIFAST III (GC Corp., Tokyo, Japan). A low-toxicity silicon adhesive (World Precision Instruments) was placed on the optical fiber to keep it clean.

After stereotaxic surgery, the mice received an intraperitoneal injection of 0.1 mg/kg buprenorphine (Otsuka Pharmaceutical Ltd.) and 10 mg/kg gentamicin (Sigma-Aldrich, St. Louis, MO) and allowed to recover for 3 weeks.

### Synthetic receptor expression in the IC for manipulating neuronal activity

Inhibitory Designer Receptors Exclusively Activated by Designer Drugs (DREADD) hM4Di were expressed in the aIC by bilateral injection of AAVdj-CaMKIIα-hM4Di-mCherry (3.2 × 10^12^ particles/mL; 250 nL per side). Excitatory DREADD hM3Dq was expressed in the aIC by bilateral injection of AAVdj-CaMKIIα-hM3Dq-mCherry (4.5 × 10^12^ particles/mL; 250 nL per side). As controls for CaMKIIα promoter-driven DREADD experiments, AAVdj-CaMKIIα-mCherry was injected into the aIC.

Behavioral experiments were performed at least 3 weeks after genetic labeling. For neuronal inhibition, mice expressing hM4Di-mCherry received an intraperitoneal injection of 5 mg/kg clozapine-N-oxide (CNO; Cayman Chemical, Ann Arbor, MI; dissolved in saline containing 1% dimethyl sulfoxide) 30 min before an intraperitoneal injection of ketamine. For neuronal activation, mice expressing hM3Dq-mCherry or mCherry (as a control) received an intraperitoneal injection of 1 mg/kg CNO 30 min before behavioral tests.

### Fiber photometry in the aIC for measuring neuronal activity

GCaMP6f was expressed in the right aIC by unilateral injection of AAVdj-CaMKIIα-GCaMP6f-P2A-nls-dTomato (1.2 × 10^12^ particles/mL; 400 nL), and a fiber cannula was implanted over the injection site (stereotaxic coordinates: +2.1 mm anterior, ± 2.6 mm lateral, and −3.5 mm ventral to the bregma). Behavioral experiments were performed at least 3 weeks after genetic labeling. For neuronal recording, a TeleFipho wireless head-stage device (dimensions: 12 × 12 × 22 mm, excitation wavelength peaked at 470 nm with a 445–490 nm filter band; emission wavelength with a 500–550 nm filter band; Nihon Bioresearch Inc.) was attached to the cannula at least 30 min before recording. The transmitter signal was sent to a digital receiver and PowerLab (ADInstruments, Sydney, Australia), an external data recording system, to be recorded in sync with the video of the behavioral performance. The signals were collected at a sampling frequency of 100 Hz. For the analysis of neural activity, a 5 min baseline was recorded for each mouse in the home cage before the first saline treatment. All signals were recorded during drug treatment and behavioral tests. Each signal was processed using a Python-based adaptive iteratively reweighted penalized least squares algorithm [43], which included signal correction and smoothing, and z-scored using signals from the 5-minuite baseline period. For nonsequential interactions, we selected events with a time difference of at least 10 s from the end of the previous investigation event [10]. To compare the aIC neuronal activity between mouse and object interactions, the mean z-score during baseline periods (3 s before interaction) was subtracted with the mean z-score during the evoked period (3 s after interaction) for each behavioral test.

### Statistical analysis

All behavioral experiments were performed, scored, and analyzed blinded to the genotype and virus injection. Before parametric tests, Levene’s test was used to compare variances using the Python statistical package scipy.stats.levene. For comparisons among three or more groups, one-way analysis of variance (ANOVA), two-way ANOVA, or two-way repeated measures ANOVA was used, where applicable. As a post hoc analysis, Tukey’s multiple comparisons test, Bonferroni’s multiple comparison test, Holm-Sidak multiple comparisons test, or Dunnett’s multiple comparisons test were used. For comparisons between two groups, the parametric two-tailed unpaired or paired t-test was used, where applicable. Spearman’s correlation analysis was used for the correlation. Detailed statistical analyses including the sample size are indicated in the figure legends. Statistical significance was set at *p* < 0.05. Statistical analyses were performed using GraphPad Prism 7.04 (GraphPad Software, San Diego, CA).

### Abbreviations for brain areas

The abbreviations for the brain areas based on the Allen Brain Reference Atlas are listed in Supplementary Table S1.

## Results

### Insular cortex activation as a key feature in (*R*)-ketamine-treated brain

Social isolation rearing from weaning is a commonly used mouse model of early life stress that results in robust behavioral and neurobiological alterations [6, 7]. Social isolation-reared mice exhibited increased immobility time in the FST, which is considered a phenotype related to the symptoms of helplessness observed in depression [44, 45]. We first examined the acute effects of subanesthetic doses (10 and 20 mg/kg) of (S)- and (*R*)-ketamine on immobility time in the FST 30 min after administration in group-reared and social isolation-reared mice. Consistent with previous reports [6, 32, 35], group-reared mice treated with (*R*)-ketamine at doses of 10 and 20 mg/kg and (*S*)-ketamine at 20 mg/kg exhibited a significant decrease in their immobility time (Supplementary Fig. S1a, b). Similar outcomes were observed in social isolation-reared mice, demonstrating a marked reduction in immobility upon treatments with (*R*)-ketamine at both 10 and 20 mg/kg, and with (*S*)-ketamine at 20 mg/kg (Supplementary Fig. S1c, d). Subsequently, we assessed their sustained effects at 20 mg/kg, by measuring immobility time in the FST 7 days after administration in social isolation-reared mice. Despite the fact that the immobility time during the second trial was considerably longer than that during the first, the decrease in immobility time prompted by both ketamine enantiomers remained evident for up to a week after administration (Supplementary Fig. S1e). The immobility time in the FST is influenced by locomotor activity of a mouse. However, the previous study has reported no significant difference in locomotor activity of wild-type mice was observed during the 10-min period starting 30 min after treatments of the ketamine enantiomers doses used in this study [46].

**Fig. 1:**
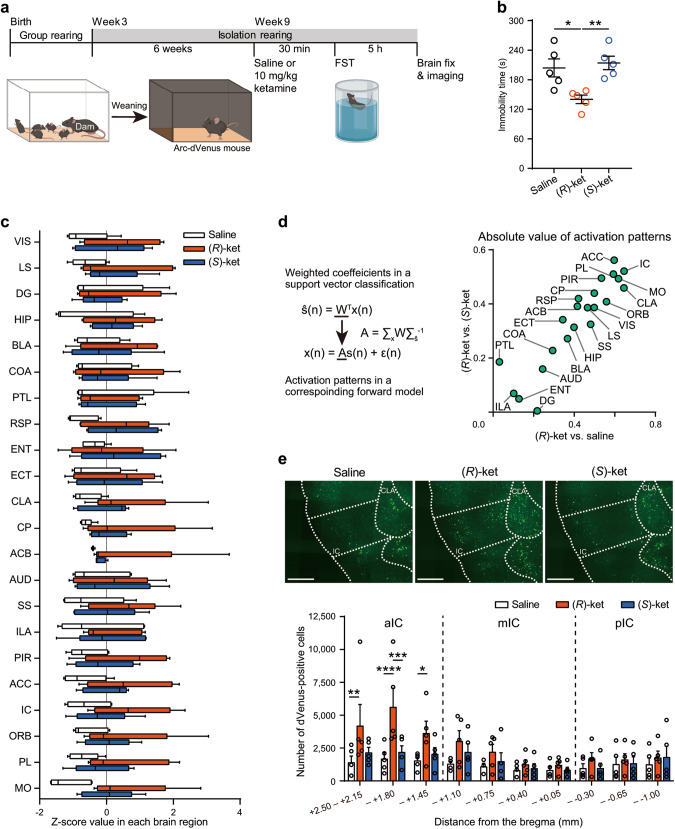
Insular cortex activation as a key feature in (*R*)-ketamine-treated brain. **a** Experimental timeline and schematic of the social isolation rearing and brain-wide IEG mapping after the forced swim test using Arc-dVenus mice. FST, forced swim test. **b** Immobility time of social isolation-reared Arc-dVenus mice treated with indicated drugs in the FST (for saline, 10 mg/kg (*R*)-ketamine, and 10 mg/kg (*S*)-ketamine; *n* = 5, in each group). Statistical analysis was performed using Levene’s test and one-way ANOVA, followed by Tukey’s multiple comparison test. Levene’s test: *p* = 0.223; one-way ANOVA: *F*(2, 12) = 8.129, *p* = 0.0059. *p < 0.05, **p < 0.01. (*R*)-ket, (*R*)-ketamine; (*S*)-ket, (*S*)-ketamine. **c** The box-whisker plot (center: median; box: 25–75th percentiles; whiskers: minimum to maximum) displays the z-score values for the number of dVenus-positive cells in each brain region. The abbreviations for the brain areas are provided in table S1. **d** The formula shows the transformation of classifier weights into corresponding activation patterns (left). The absolute values of the activation patterns of brain regions are plotted in the two indicated classifications. **e** Representative coronal images of the anterior insular cortex (aIC) of Arc-dVenus mice treated with indicated drugs (top). Scale bar, 500 µm. The anteroposterior distribution of dVenus-positive cells in the IC is shown in the bottom panel. mIC, the middle IC; pIC, the posterior IC. Statistical analysis was performed using Two-way ANOVA, followed by Tukey’s multiple comparison test: spatial distribution, *F*(9, 120) = 4.378, *p* < 0.0001; treatment, *F*(2, 120) = 14.12, *p* < 0.001; interaction, *F*(18, 120) = 1.191, *p* = 0.2791. **p < 0.01, ***p < 0.001, ****p < 0.0001. Data are presented as the mean ± standard error of the mean (s.e.m.).

To investigate neuronal responses to drug treatments, brain-wide mapping of IEGs is a valuable tool [41, 47]. Due to the more pronounced differences in the effects of ketamine enantiomers at 10 mg/kg in social isolation-reared mice shown in Supplementary Fig. S1, we generated social isolation-reared mice using Arc-dVenus mice as IEG reporter mice and then investigated the effects of the 10 mg/kg ketamine enantiomers on their immobility time in the FST (Fig. 1a, b) [48]. Consistent with the results in wild-type mice, the immobility time in the FST of Arc-dVenus mice, assessed using brain-wide mapping of IEG, significantly decreased when administered with 10 mg/kg (*R*)-ketamine, while no significant change was observed with 10 mg/kg (*S*)-ketamine compared to saline (Fig. 1b). To investigate the differential effects of (*S*)- or (*R*)-ketamine on neuronal responses, we captured three-dimensional reconstructed brain images of social isolation-reared Arc-dVenus mice 5 h after the FST using the high-speed whole-brain imaging system, FAST [39, 40]. We then counted dVenus-positive cells in 22 brain regions based on morphological characteristics as brain-wide IEG maps. No significant differences were observed between the drug treatments in these brain regions (Fig. 1c). To identify key brain regions responsive to (*R*)-ketamine treatment, we analyzed the brain-wide IEG maps using linear support vector classification and calculated the corresponding activation patterns to discriminate between 10 mg/kg (*R*)-ketamine treatment and 10 mg/kg (*S*)-ketamine or saline treatment in an unbiased manner. The scatterplot of the absolute values of the corresponding activation patterns indicated that the IC, anterior cingulate cortex (ACC), and prelimbic cortex predominantly contributed to the two discriminations (Fig. 1d), among which the IC showed the strongest contribution to the discrimination between the (*R*)-ketamine and saline treatments.

To investigate the spatial distribution of dVenus-positive cells in the IC, which has a relatively long anteroposterior axis (approximately +2.5 to –1.0 mm across the bregma), we examined the number of dVenus-positive cells in different parts of the IC [49]. (*R*)-ketamine treatment significantly increased the number of dVenus-positive cells in the aIC (+2.50 to +1.45 mm anterior to the bregma) (Fig. 1e).

### Attenuation of (*R*)-ketamine’s effect on immobility time via aIC inhibition

**Fig. 2:**
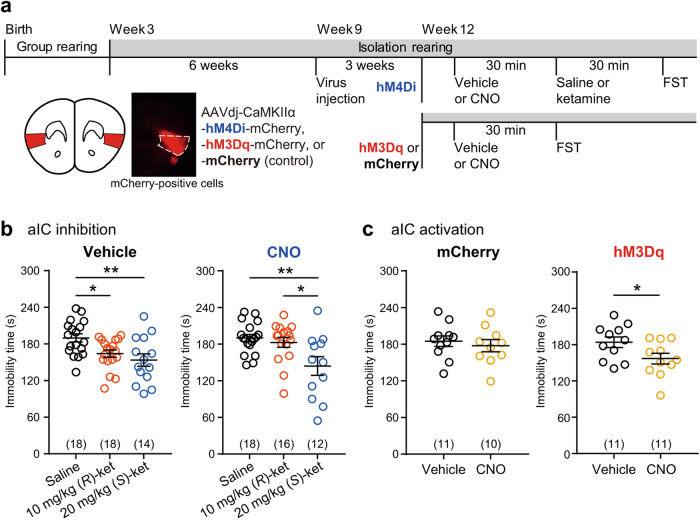
Attenuation of (*R*)-ketamine’s effect on immobility time via aIC inhibition. **a** Experimental timeline and schematic of chemogenetic manipulation of the aIC in social isolation-reared mice and a schematic and representative image illustrating the selective transduction of hM4Di-mCherry at the injection site (highlighted in red). CNO, clozapine-N-oxide. **b** Immobility time of hM4Di-mCherry-expressing mice treated with 10 mg/kg (*R*)-ketamine or 20 mg/kg (*S*)-ketamine in the FST. Statistical analysis was performed using Levene’s test and one-way ANOVA, followed by Bonferroni’s multiple comparison test. Levene’s test: *p* = 0.153 (vehicle), *p* = 0.012 (CNO); one-way ANOVA: Vehicle, *F*(2, 47) = 6.198, *p* = 0.0041; CNO, *F*(2, 43) = 6.044, *p* = 0.0049. *p < 0.05, **p < 0.01. **c** Immobility time of hM3Dq-mCherry- or mCherry-expressing mice treated with vehicle or CNO in the FST. The number in parentheses indicates the number of mice used in the experiment. Statistical analysis was performed using Levene’s test and unpaired *t* test. Levene’s test: *p* = 0.744 (mCherry), *p* = 0.828 (hM3Dq). *p < 0.05. Data are presented as the mean ± s.e.m.

Next, we investigated the involvement of the aIC in the effect of ketamine enantiomers on immobility time in social isolation-reared mice via chemogenetic manipulations of the aIC (Fig. 2). Previous research indicates that the long-range projection neurons in the aIC are predominantly CaMKIIα-positive cells [49]. Furthermore, in our experiments with Arc-dVenus mice, it was observed that dVenus-positive cells in these mice exhibit high levels of CaMKIIα expression [41]. To suppress neuronal activity in these CaMKIIα-positive cells of the aIC, we expressed the synthetic inhibitory receptor hM4Di in social isolation-reared mice (Fig. 2a). The period of social isolation rearing was extended by 3 weeks compared to the experiment described above due to surgery and its recovery, but the extended period had little effect on behaviors. Mice expressing hM4Di-mCherry treated with vehicle 30 min before 20 mg/kg (*S*)-ketamine or 10 mg/kg (*R*)-ketamine treatment showed a reduction in the immobility time (Fig. 2b). However, in the group of mice that received CNO, a commonly used ligand for chemogenetic receptors, the reduction in immobility time induced by (*R*)-ketamine was suppressed, whereas the reduction of immobility time induced by (*S*)-ketamine was unaffected (Fig. 2b). We then investigated the effect of chemogenetic activation of the aIC on immobility time in the FST using a synthetic excitatory receptor hM3Dq. Mice expressing hM3Dq-mCherry showed a decrease in immobility time upon the administration of CNO, whereas mice expressing mCherry did not show a decrease in immobility time even after CNO administration (Fig. 2c). These results indicate that aIC activation is involved in the effect of (*R*)-ketamine, but not (*S*)-ketamine.

To validate the effect of CNO on cells expressing hM4Di-mCherry in the aIC, we administered CNO 30 min before (*R*)-ketamine administration to mice expressing hM4Di-mCherry. Administration of CNO led to a notable reduction in c-Fos expression in mCherry-positive cells within the aIC. In addition, immunohistochemical analysis confirmed the high prevalence of CaMKIIα-positive cells in the aIC (Supplementary Fig. S2a–e). Furthermore, in mice expressing hM3Dq-mCherry, we found that CNO treatment alone increased c-Fos expression in the aIC (Supplementary Fig. S2f–i). These results provide robust evidence for the functional efficacy of DREADD manipulations in these neuronal populations.

### Boost of aIC neuronal responses to social investigation by (*R*)-ketamine

**Fig. 3:**
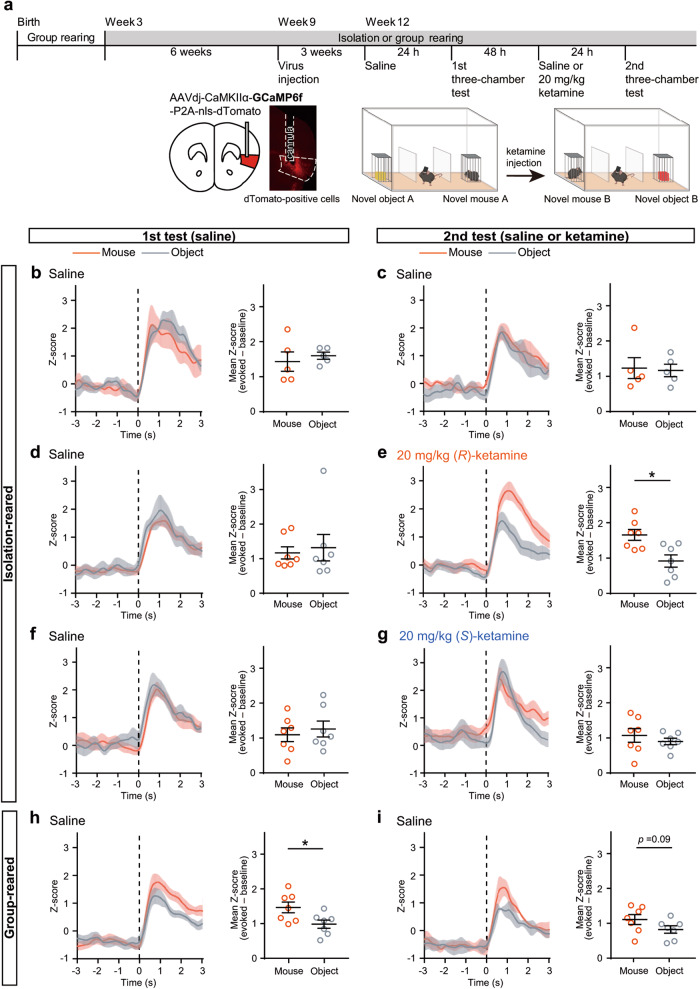
Boost of aIC neuronal responses to social investigation by (*R*)-ketamine. **a** Experimental timeline and schematic of fiber photometry recording in social isolation- or group- reared mice during the three-chamber test. Neuronal activity of the aIC in social isolation-reared mice was observed during nonsequential interactions with a novel mouse or object in the 1st-3-chamber test with saline treatment (**b**, **d**, and **f**) and the 2nd-3-chamber test with drug treatment (**c**, **e**, and **g** for saline, 20 mg/kg (*R*)-ketamine, and 20 mg/kg (*S*)-ketamine; *n* = 5, 7, and 7, respectively). The right graphs show the quantification of social (orange) or object (gray) contact-evoked normalized GCaMP6f signals (mean evoked z-score of 0‒3 s at post-contact subtracted by the mean baseline z-score of −3‒0 s at pre-contact). Statistical analysis was performed using paired *t* test. *p < 0.05. Neuronal activity of the aIC in group-reared mice was observed in nonsequential interactions with a novel mouse or object in the 1st-3-chamber test (**h**) and 2nd-3-chamber test (**i**) with saline treatment (*n* = 7). The right graphs show the quantification of social (orange) or object (gray) contact-evoked normalized GCaMP6f signals (mean evoked z-score of 0‒3 s at post-contact subtracted by the mean baseline z-score of −3–0 s at pre-contact). Dashed lines indicate the onset of contact. Statistical analysis was performed using paired *t* test. *p < 0.05. Data are presented as the mean ± s.e.m.

Chronic social isolation rearing has been reported to impair social functions such as social monitoring abilities [8–10]. Given the involvement of aIC activity in social cognition including social investigation and memory [50, 51], we measured aIC calcium activity during social investigation in social isolation-reared mice treated with (*S*)- or (*R*)-ketamine using fiber photometry in the three-chamber test twice (1st- and 2nd-three-chamber tests) (Fig. 3). In this study, to control for potential variations arising from dose differences, we consistently used a dose of 20 mg/kg, as both ketamine enantiomers showed effects in the FST (Supplementary Fig. S1). The 1st-three-chamber test was performed on the next day after all mice were treated with saline. We observed a similar increase in aIC activity immediately after contact with the object or mouse during the 1st-three-chamber test (Fig. 3b, d, and f). During the 2nd-three-chamber test, aIC activity showed a similar level of increase during contact with the object or mouse on the day after saline or (*S*)-ketamine treatment, as observed in the 1st-three-chamber test (Fig. 3c, g). In contrast, in social isolation-reared mice treated with (*R*)-ketamine, aIC activity immediately after contact with the mouse showed a significant increase compared to that after contact with the object (Fig. 3e and Supplementary Fig. S3a). Furthermore, the increase in aIC activity following contact with the mouse showed a significant positive correlation with the subsequent duration of contact with the mouse (Supplementary Fig. S3b). Group-reared mice showed a significant increase in aIC activity in response to the mouse compared to the response to the object during the 1st-three-chamber test, and a similar trend was observed in the 2nd-three-chamber test (Fig. 3h, i). These results suggest that (*R*)-ketamine treatment may restored typical aIC functionality.

Prolonged social isolation rearing is known to elicit fear responses toward unfamiliar mice [52]. To investigate the potential influence of the fear responses on aIC activity, we reintroduced mice to group rearing for 3 weeks following 6 weeks of social isolation. We then assessed the aIC calcium activity of these re-socialized mice, treated with (*R*)-ketamine at 20 mg/kg, during the three-chamber tests using fiber photometry (Supplementary Fig. S4). Consistent with the aIC responses observed in social isolation-reared mice (Fig. 3), re-socialized mice did not display a differential increase in aIC activity immediately after contact with a mouse or an object after saline treatment (Supplementary Fig. S4b–d). In contrast, under (*R*)-ketamine treatment, aIC responses were significantly heightened upon interaction with an unfamiliar mouse as opposed to an object (Supplementary Fig. S4e). After the three-chamber tests, we further investigated whether (*R*)-ketamine was effective not only in distinguishing between a mouse and an object, but also in distinguishing between familiar and unfamiliar mice. We explored the variations in aIC responses in a social recognition test in which the object was replaced with a cage mate. While saline-treated mice exhibited similar aIC responses to both unfamiliar mice and cage mates, the (*R*)-ketamine-treated group exhibited significantly stronger responses to unfamiliar mice than to cage mates (Supplementary Fig. S4f, g).

To investigate the mechanisms underlying aIC activation by (*R*)-ketamine, we performed whole-cell voltage clamp patch recordings of pyramidal neurons in the aIC from brain slices of 3-week-old mice. We found no significant current change when the membrane potential was maintained at −80 mV during (*R*)-ketamine treatment (Supplementary Fig. S5). This result suggested that the effect of (*R*)-ketamine on the pyramidal neuron is not through the direct ligand-gated action nor conductance change at the resting membrane potential in the aIC. In addition, the rapid antidepressant effects of ketamine are thought to target BDNF–TrkB signaling, as BDNF is known to increase excitatory evoked release and attenuate inhibitory neurotransmission [46, 53, 54]. To investigate whether (*R*)-ketamine activates excitatory neurons in the aIC during social cognition by modulating BDNF–TrkB signaling, we performed a pharmacological study of aIC neural activity changes using the three-chamber tests. Inhibition of the BDNF receptor TrkB by ANA-12 treatment did not diminish the effect of (*R*)-ketamine in social isolation-reared mice, which exhibited increased neural activity when interacting with unfamiliar mice compared to objects (Supplementary Fig. S6). This result indicated that the ability of (*R*)-ketamine to restore aIC function may not involve modulation of BDNF–TrkB signaling.

### Restoration of social cognitive deficits through (*R*)-ketamine-induced activation of the aIC

Given that (*R*)-ketamine restored aIC responses related to social investigation, we next conducted a 5-trial social memory test twice, using saline and (*R*)-ketamine. As the first step, in both the first and second memory tests, we observed that group-reared mice exhibited a robust decrease in investigation time toward the same intruder mouse during the first four trials, indicating the formation of social memory (Supplementary Fig. S7). During the 5th trial, we observed an increase in investigation time toward the novel intruder mouse, indicating a social novelty response. In group-reared mice, we detected significant formation of social memory and social novelty response during both the first and second tests (Supplementary Fig. S7b, c). These changes in investigation time were consistent with previously reported patterns of social memory formation [38].

**Fig. 4:**
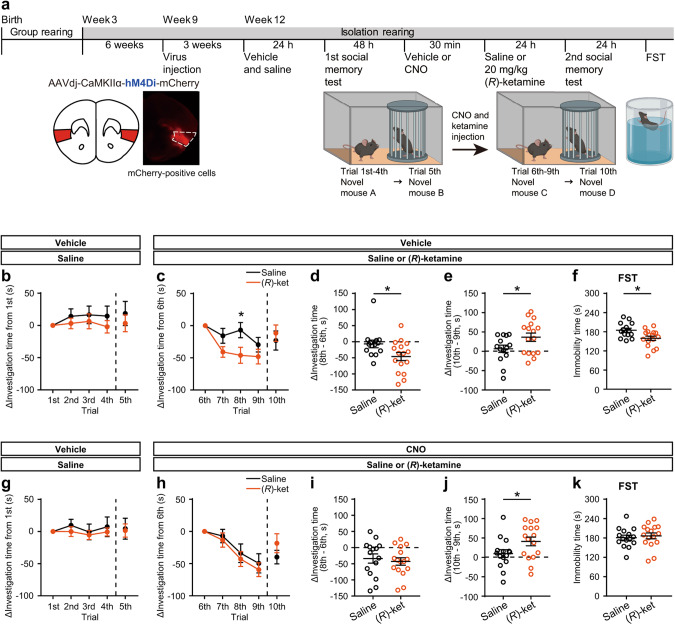
Restoration of social cognitive deficits through (*R*)-ketamine-induced activation of the aIC. **a** Experimental timeline and schematic of the social memory test using social isolation reared-mice with or without chemogenetic aIC inhibition and representative image of hM4Di-mCherry transduction at the injection site (highlighted in red). **b** Difference of investigation time to a familiar mouse between 1st trial and indicated trial (2nd to 4th trial) and to a novel mouse (5th trial) in the 1st social memory test 24 h after vehicle and saline treatments. **c**–**e** Difference of investigation time to a familiar mouse between 6th trial and indicated trial (2nd to 4th trial) and to a novel mouse (10th trial) in the 2nd social memory test 24 h after vehicle and saline or 20 mg/kg (*R*)-ketamine treatments (for saline and (*R*)-ketamine; *n* = 14 and 16, respectively). Statistical analysis was performed using two-way repeated measured ANOVA, followed by Holm-Sidak multiple comparison test (**c**): time, *F*(4, 112) = 7.023, *p* < 0.0001; treatment, *F*(1, 28) = 1.57, *p* = 0.2206; interaction, *F*(4, 112) = 3.36, *p* = 0.0123, and using Levene’s test and unpaired *t* test (**d**, **e**). Levene**’**s test: *p* = 0.207 (**d**), *p* = 0.179 (**e**); unpaired *t* test, *p < 0.05. **f** Immobility time of hM4Di-mCherry-expressing mice in the FST 48 h after drug treatments. Statistical analysis was performed using Levene’s test and unpaired *t* test (**f**). Levene’s *t*est: *p* = 0.707; unpaired *t* test, *p < 0.05. **g** Difference of investigation time to a familiar mouse between 1st trial and indicated trial (2nd to 4th trial) and to a novel mouse (5th trial) in the 1st social memory test 24 h after vehicle and saline treatments. **h**–**j** Difference of investigation time to a familiar mouse between 6th trial and indicated trial (2nd to 4th trial) and to a novel mouse (10th trial) in the 2nd social memory test 24 h after CNO and saline or 20 mg/kg (*R*)-ketamine treatments (for saline and (*R*)-ketamine; *n* = 15 and 16, respectively). Statistical analysis was performed using two-way repeated measured ANOVA: time, *F*(4, 116) = 15.03, *p* < 0.0001; treatment, *F*(1, 29) = 0.004067, *p* = 0.9496; interaction, *F*(4, 116) = 1.352, *p* = 0.2550, and using Levene’s test and unpaired *t* test (**i**, **j**). Levene’s test: *p* = 0.276 (**i**), *p* = 0.221 (**j**); unpaired *t* test, *p < 0.05. **k** Immobility time of hM4Di-mCherry-expressing mice in the FST 48 h after drug treatments. Statistical analysis was performed using Levene’s test and unpaired *t* test. Levene’s test: *p* = 0.441. Black lines, mouse treated with saline in both 1st and 2nd social memory test; Orange line, mouse treated with saline in 1st and (*R*)-ketamine in 2nd social memory test. Data are presented as the mean ± s.e.m.

To investigate whether the aIC is involved in the effects of 20 mg/kg (*R*)-ketamine on social memory, we performed two rounds of 5-trial social memory tests followed by the FST in social isolation-reared mice expressing hM4Di-mCherry (Fig. 4). In the initial social memory test, there was no significant change in investigation time when social isolation-reared mice, treated with vehicle and saline, explored a novel mouse in the 1st and 5th trials and repeatedly explored a familiar mouse from the 2nd to 4th trials (Fig. 4b, g). The total investigation time of these social isolation-reared mice in the 1st trial was lower compared to that of group-reared mice [social isolation-reared mice, 59.77 ± 7.25 s; group-reared mice, 98.72 ± 12.03 s (mean ± s.e.m.)]. It is believed that the intense fear caused by prolonged social isolation leads to extended acclimation time for mice when encountering unfamiliar counterparts. In the subsequent social memory test performed 2 days after the initial one, social isolation-reared mice treated with vehicle and saline displayed an increase in investigation time from the 5th to the 6th trial [saline, 5th trial, 76.37 ± 15.61 s, 6th trial, 128.13 ± 13.03 s (mean ± s.e.m.)] and a progressive decrease in investigation time to a familiar mouse from the 7th trial through the 9th trial (Fig. 4c). (*R*)-ketamine-treated mice exhibited a similar increase in investigation time from the 5th to the 6th trial as observed in saline-treated mice [(*R*)-ketamine, 5th trial, 66.57 ± 14.33 s, 6th trial, 129.37 ± 13.70 s (mean ± s.e.m.)]. In addition, (*R*)-ketamine treatment enhanced the decline in investigation time to a familiar mouse, with a significant decrease in the 8th trial compared to the saline treatment (Fig. 4d). In the 10th trial, the investigation time for a novel mouse showed little difference in the saline-treated mice compared to the 9th trial, whereas a significant increase was observed in the (*R*)-ketamine-treated mice (Fig. 4e). In contrast, in social isolation-reared mice treated with CNO, which inhibits aIC activation, no significant difference in investigation time to a familiar mouse was observed from the 7th to 9th trials in mice treated with saline compared to those treated with (*R*)-ketamine (Fig. 4h, i). However, mice treated with CNO still exhibited a significant increase in investigation time between the 9th and 10th trials as the social novelty responses by (*R*)-ketamine treatment (Fig. 4j). These data indicated (*R*)-ketamine restored the social cognitive deficits in social isolation-reared mice, in part, through the aIC activation.

To confirm the effects of chemogenetic inhibition of the aIC and to investigate whether the sustained effect of (*R*)-ketamine on immobility time in the FST is mediated by aIC activation, we conducted the FST the day after the 2nd 5-trial social memory test. As the effects of aIC inhibition on the acute effects of (*R*)-ketamine shown in Fig. 2, the reduction in immobility time 48 h after (*R*)-ketamine treatment was abolished by chemogenetic inhibition of the aIC (Fig. 4f, k). These results demonstrated that aIC activation immediately after (*R*)-ketamine administration is involved not only in a partial restoration of social cognition but also in sustaining its effect on immobility time in the FST.

## Discussion

Our study aimed to investigate the neural mechanisms underlying the differential effects of ketamine enantiomers, (*S*)- and (*R*)-ketamine, on behavioral and neuronal activity abnormalities caused by chronic social isolation rearing. Our results indicated that (*R*)-ketamine induced the unique activation of the aIC, which promotes the formation of social memory. While several studies have compared the effects of ketamine enantiomers in animal models, revealing differences in therapeutic effect and abuse potential at the molecular level, in specific brain regions [55, 56], the cellular and circuit-level mechanisms remain obscure. Therefore, our findings provide additional insights into the therapeutic potential of ketamine at the neuronal activity level, rather than its potential for abuse.

The aIC plays a crucial role in emotional regulation [57, 58] and valence processing [59, 60], including pleasurable experiences [61, 62]. Certain parts of the aIC are activated during social investigation [50] and are responsible for social novelty recognition [51]. Our study found that neural activity in the aIC increased during social contact in group-reared mice; however, this response was absent in social isolation-reared mice. Meanwhile, (*R*)-ketamine could restore aIC neuronal activity in social isolation-reared mice during social contact. A recent study using CaMKIIα-Cre mice elucidated the insular cortex’s connections to various downstream brain regions [49]. A key finding is that CaMKIIα-positive cells in the aIC predominantly project to the striatum. Specifically, mouse connectivity data from the Allen Brain Atlas highlight projections from the aIC to the ventral striatum, including the nucleus accumbens. This suggests a potential role of the aIC-ventral striatum pathway in modulating responses to pleasurable social stimuli, although additional research is necessary to validate this hypothesis. In addition, in clinical studies, racemic ketamine increased neural activation in the IC of healthy volunteers [63, 64]. These findings suggest that (*R*)-ketamine induces positive emotions during social contact in mice and may improve social deficits in humans by activating the aIC.

A recent brain-wide mapping study on another IEG, c-Fos, has reported that racemic ketamine activates numerous brain regions, with the largest change in c-Fos expression was observed in the ACC [65]. Our data, as shown in Fig. 1, revealed that (*R*)-ketamine activates multiple brain regions including the ACC, implying that ACC activation by racemic ketamine is influenced by (*R*)-ketamine. The results of the two discriminant analyses of brain-wide IEG maps suggest that the effect of (*R*)-ketamine is not limited to the activation of the IC but also involves other regions such as the ACC and CLA. Particularly, the IC, ACC, and CLA form reciprocal neural circuits with each other and together constitute the salience network [66–69]. In patients with major depression disorder, racemic ketamine normalizes the connectivity between the insular and salience networks [70], which are involved in processing external stimuli and play central roles [71]. These findings suggest that (*R*)-ketamine restores cognitive function in response to social stimuli by inducing functional recovery of the salience network, potentially differing from the effects of (*S*)-ketamine and revealing the underlying mechanisms of social cognition, although further investigation is required.

In the social memory test conducted on mice reared in chronic social isolation, these mice exhibited brief periods of social contact, with no observable changes in behavior across the first to fifth trials. This short contact time was likely due to the mice encountering intruder mice for the first time post-isolation, resulting in avoidance behaviors and the maintenance of their distance. In other research protocols [8, 9], a period of group rearing was included after chronic social isolation to provide an acclimation period for these mice. During the 6th trial, the fear response toward unfamiliar mice decreased, contact time increased, and the trial-dependent decrease in social cognition effect was evaluated from the 6th to 10th trials. Furthermore, administering ketamine or saline just before the 6th trial did not result in any difference in contact time, indicating that ketamine does not affect the acclimation or fear reduction of mice, at least in the 6th trial.

The prevalence of various mental illnesses, such as depression, has been increasing worldwide in 2021, following restrictions on social activities due to the coronavirus disease 2019 pandemic. This increase is attributed not only to inflammatory factors, such as respiratory diseases and pneumonia caused by viral infections, but also to psychological factors resulting from social isolation [4, 72]. Social isolation, particularly from a young age, has been shown to increase aggression and anxiety levels and affect basal emotions. Additionally, it decreases sociality, thus, negatively impacting an individual’s mental maturation. Therefore, even after the end of social isolation, mental symptoms caused by social isolation may persist, and the incidence of mental illnesses is expected to continue to increase. (*R*)-ketamine, the focus of this study, is noted for its potential antidepressant effects, although clinical trials are currently ongoing and its efficacy is still controversial. In our study, we observed differential improvements in social cognitive function and behavior associated with helplessness by ketamine enantiomers, indicative of the underlying distinct neural mechanisms. These improvements indicate complex interactions beyond conventional frameworks of mood and cognition. Social cognitive impairments, common features of psychiatric disorders such as depression and schizophrenia, are generally less severe in patients with depression than in those with schizophrenia [73, 74]. Therefore, our findings on the ability of (*R*)-ketamine to ameliorate social cognitive deficits may contribute to the development of treatments for various mental disorders that share similar social cognitive deficits.

## Limitations of the study

We conducted brain-wide IEG mapping in socially isolated Arc-dVenus mice following the FST, to identify key brain regions influenced by (*R*)-ketamine. However, crucial experimental limitations must to be considered. First, the neural activity effects triggered by the FST itself cannot be excluded. Additionally, contrasting our results with experiments involving group-reared mice could provide deeper insights into the impact of social isolation rearing. Furthermore, while our current study primarily emphasized the aIC, the effects of (*R*)-ketamine may also manifest in regions such as the ACC and CLA. Investigating this through circuit-selective neuronal activity manipulations and measurements, considering the anatomical interconnections of these regions, is pivotal for a more detailed understanding of their precise neural mechanisms.

### Supplementary information

Supplemental Information

## Data Availability

The data that support the findings of this study are available upon request from the corresponding author [AK]. All original code for machine learning in this study has been deposited at Zenodo (accession codes, 10.5281/zenodo.10512326) and are publicly available as of the data of publication. Supplementary information is available at MP’s website.
