# Supplementary material for: (R)-ketamine restores anterior insular cortex activity and cognitive deficits in social isolation-reared mice
Source: Mol Psychiatry. 2024 Feb 23;29(5):1406–16. doi: 10.1038/s41380-024-02419-6 (PMC11189812; doi:10.1038/s41380-024-02419-6)
Supplement: Supplementary file 1 — Supplemental Information [file 41380_2024_2419_MOESM1_ESM.pdf]

Supplementary Information for

(*R*)-ketamine restores anterior insular cortex activity and cognitive deficits in social isolation-reared mice

Yokoyama R et al.

This PDF files includes:

Supplementary Material and Methods
Supplementary References
Supplementary Fig. S1 to S7
Supplementary Table S1

Supplemental material and Methods

Materials

ANA-12 (Cayman Chemical) was dissolved in vehicle of 1% dimethyl sulfoxide in phosphate-buffered saline (PBS; 1.5 mM KH_2PO4, 8.1 mM NA_2HPO4, 137 mM NaCl, and 2.7 mM KCl (pH 7.4)) just before use.[1]

Immunohistochemistry

hM4Di-mCherry or hM3Dq-mCherry was expressed in the right aIC in group-reared mice by unilateral injection of AAVdj-CaMKIIα-hM4Di-mCherry or AAVdj-CaMKIIα-hM3Dq-mCherry (250 nL per side). For the hM4Di group, we administered 5 mg/kg CNO, followed by 10 mg/kg of (*R*)-ketamine 30 minutes later. The FST was conducted 30 minutes after the (*R*)-ketamine administration, and fixation was performed 1.5 hours after the FST. For the hM3Dq group, we administered 1 mg/kg CNO and then proceeded to fixation after 1.5 hours. Paraformaldehyde-fixed brains expressing hM4Di-mCherry or hM3Dq-mCherry were cryoprotected for several days in 20 to 30% sucrose dissolved in PBS. Subsequently, tissue blocks containing the aIC were sliced into 30-µm-thick coronal sections using a Leica CM1860 cryostat (Leica Microsystem Ltd., Germany). The sections were then subjected to a free-floating staining technique as described previously[2] with minor modifications. Briefly, the sections were blocked with 5% normal gout serum albumin (Thermo Fisher Scientific, Waltham, MA) in PBS containing 0.3% Triton X-100 (FUJIFILM Wako Pure Chemical Corp) for 1 h at room temperature, incubated with mouse anti-CaMKIIα monoclonal antibody (1:50 dilution; catalog number sc-13141; Santa Cruz Biotechnology, Santa Cruz, CA) and/or rabbit anti-c-Fos antibody (1:500 dilution; catalog number #2250-s; Cell Signaling Technology, Beverly, MA) overnight at 4°C and then with Alexa 405-conjugated goat anti-mouse IgG secondary antibody (1:1,000 dilution; catalog number A-48255; Life Technologies, Carlsbad, CA) and/or Alexa 488-conjugated goat anti-rabbit IgG secondary antibody (1:1,000 dilution; catalog number A-11008; Life Technologies) for 1 h at room temperature. All images of immunostained sections were obtained with a fluorescence microscope (BZ-X100; Keyence, Osaka, Japan).

Behavioral tests

Social recognition test

For re-socialization after 6 weeks social isolation, isolated-reared mice were co-housed with 2 or 3 male mice that were a week younger.

The three-chamber test was performed as previously reported.[3] The test mouse was placed in the central chamber of an opaque polyvinyl chloride box (length × width × height: 42 × 50 × 30 cm) divided into three interconnected chambers under illumination of 400 lx (measured in the center zone). Clear partitions with openings allow the mouse to freely move between chambers. After a 90 min habituation period, an unfamiliar male mouse of the same strain but 1 week younger, along with a cage-mate mouse, was introduced into separate wire-mesh intruder boxes (length × width × height: 10 × 6.5 × 20 cm). The test mouse explored both the intruder mouse and object for 10 min. "Mouse interaction" and "object interaction" were defined as the test mouse's nose contact with each interaction box.

Electrophysiology

The whole-cell patch recordings were performed using brain slices from mice, and the preparation of acute slices followed the method as described previously.[4] Three-weeks-old male mice were anesthetized with isoflurane and then decapitated. Coronal brain slices of 350 µm thickness were prepared in ice-cold *N*-methyl-D-glucamine (NMDG) artificial cerebrospinal fluid (aCSF) containing 92 mM NMDG, 2.5 mM KCl, 1.2 mM NaH_2PO_4, 30 mM $NaHCO_3$, 20 mM HEPES, 25 mM glucose, 5 mM sodium ascorbate, 2 mM thiourea, 3 mM sodium pyruvate, 10 mM $MgSO_4$, and 0.5 mM $CaCl_2$ (adjusted pH to 7.4 using HCl). These slices were then incubated in a HEPES holding solution containing 92 mM NaCl, 2.5 mM KCl, 1.2 mM NaH_2PO_4, 25 mM $NaHCO_3$, 20 mM HEPES, 25 mM glucose, 5 mM sodium ascorbate, 2 mM thiourea, 3 mM sodium pyruvate, 2 mM $MgSO_4$, and 2 mM $CaCl_2$ (adjusted pH to 7.4 using NaOH) for more than 1 hour at room temperature. The slices were transferred to a recording chamber and superfused with aCSF at

33 °C, which consisted of 124 mM NaCl, 2.5 mM KCl, 1.2 mM NaH_2PO_4, 24 mM $NaHCO_3$, 5 mM HEPES, 12.5 mM glucose, 2 mM $MgSO_4 \cdot 7H_2O$, 2 mM $CaCl_2$ (adjusted pH to 7.4 using NaOH). All solutions used for electrophysiology were equilibrated with 95% O_2/5% CO_2. Recordings were performed from pyramidal neurons in the aIC using a MultiClamp 700A amplifier and sampled at 50 kHz with a Digidata1550A A/D converter. Data acquisition and analysis were performed using pClamp 11 software (Molecular Devices, San Jose, CA). The pipette resistance was 2-5 MΩ. The intracellular solution contained the following composition: 140 mM K-gluconate, 5 mM KCl, 10 mM HEPES, 2 mM Mg-ATP, 0.3 mM Na_2-GTP, 10 mM disodium phosphocreatine, and 0.2 mM EGTA (adjusted to pH 7.3 using KOH). The membrane potential was held at -80 mV in the voltage-clamp mode. For (*R*)-ketamine treatment, perfusate was switched with 10 μM (*R*)-ketamine-containing aCSF after basal recording for 1 min.[5] We calculated the current from 0 to 1 min as a basal current and from 5 to 6 min as a current after treatment of (*R*)-ketamine.

Supplementary References

1. Yang C, Shirayama Y, Zhang JC, Ren Q, Yao W, Ma M *et al.* R-ketamine: a rapid-onset and sustained antidepressant without psychotomimetic side effects. *Translational Psychiatry* 2015; **5**(9)**:** e632.

2. Niu M, Kasai A, Tanuma M, Seiriki K, Igarashi H, Kuwaki T *et al.* Claustrum mediates bidirectional and reversible control of stress-induced anxiety responses. *Science Advances* 2022; **8**(11)**:** eabi6375.

3. Ago Y, Hasebe S, Nishiyama S, Oka S, Onaka Y, Hashimoto H *et al.* The Female Encounter Test: A Novel Method for Evaluating Reward-Seeking Behavior or Motivation in Mice. *International Journal of Neuropsychopharmacology* 2015; **18**(11)**:** 1–12.

4. Ting JT, Lee BR, Chong P, Soler-Llavina G, Cobbs C, Koch C *et al.* Preparation of Acute Brain Slices Using an Optimized *N*-Methyl-D-glucamine Protective Recovery Method. *Journal of Visualized Experiments* 2018; (132).

5. Zanos P, Moaddel R, Morris PJ, Georgiou P, Fischell J, Elmer GI *et al.* NMDAR inhibition-independent antidepressant actions of ketamine metabolites. *Nature* 2016; **533**(7604)**:** 481-486.

Supplementary Fig. S1

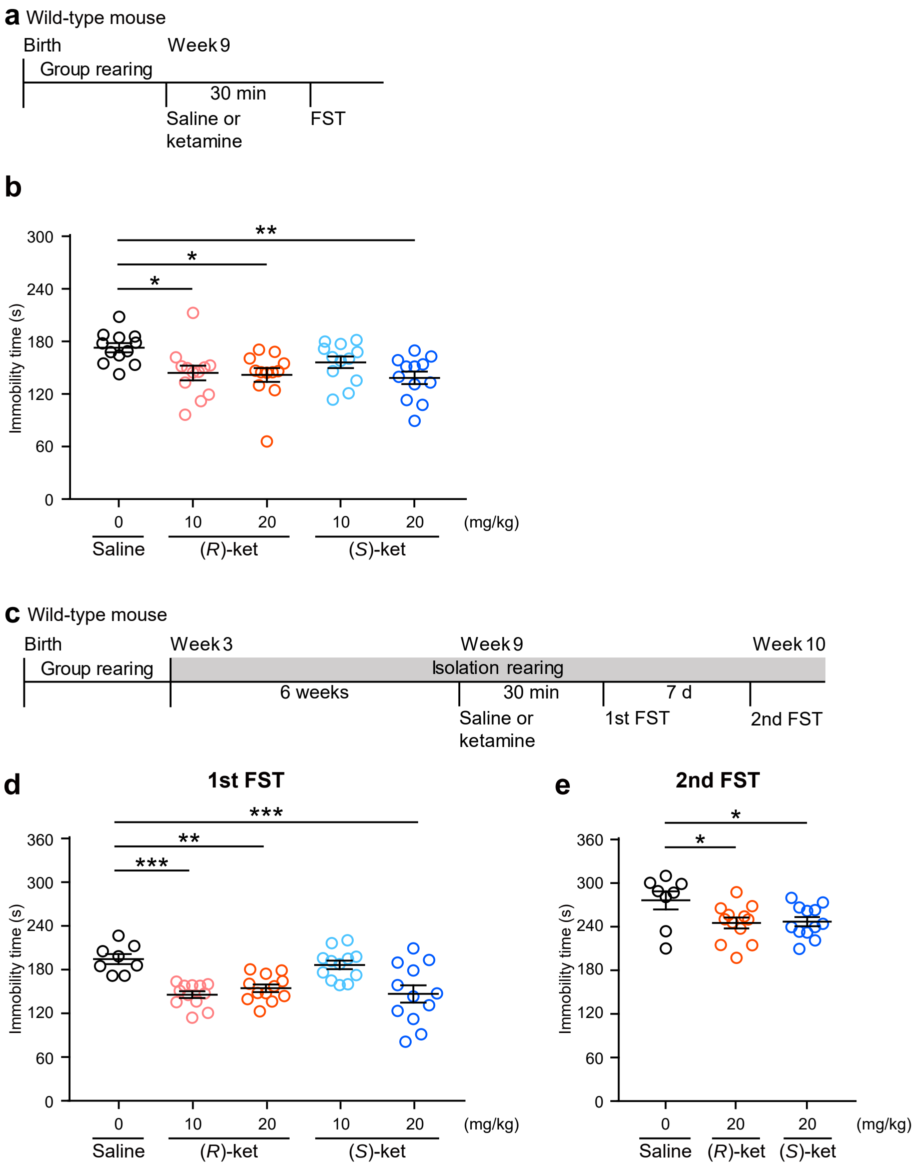

Fig. S1. Immobility time in social isolation-reared mice treated with ketamine in the FST. (**a**) Experimental timeline of forced swim test using group-reared mice treated with ketamine enantiomers. FST, forced swim test. (**b**) Immobility time of group-reared mice treated with indicated drugs in the FST (n = 12, in each group). (*R*)-ket, (*R*)-ketamine; (*S*)-ket, (*S*)-ketamine. Statistical analysis was performed using Levene´s test and one-way ANOVA, followed by Dunnett´s multiple comparison test. Levene´s test: p = 0.927; one-way ANOVA: F(4, 55) = 3.903, p = 0.007 (b). *p < 0.05, **p < 0.01. (**c**) Experimental timeline of repeated FST using social isolation-reared mice to evaluate the effects and persistence of ketamine enantiomers. (**d and e**) Immobility time of mice in the FST 30 minutes (d) and 1 week (e) after treatment with the indicated drugs (for saline, (*R*)-ketamine, and (*S*)-ketamine; n = 8, 12, and 12, respectively). Statistical analysis was performed using Levene´s test and one-way ANOVA, followed by Dunnett´s multiple comparison test. Levene´s test: p = 0.004 (d), p = 0.412 (e); one-way ANOVA; F(4, 51) = 8.840, p = 0.007 (d); F(2, 29) = 3.787, p = 0.007 (e). *p < 0.05, **p < 0.01, ***p < 0.001. Data are presented as the mean ± s.e.m.

Supplementary Fig. S2

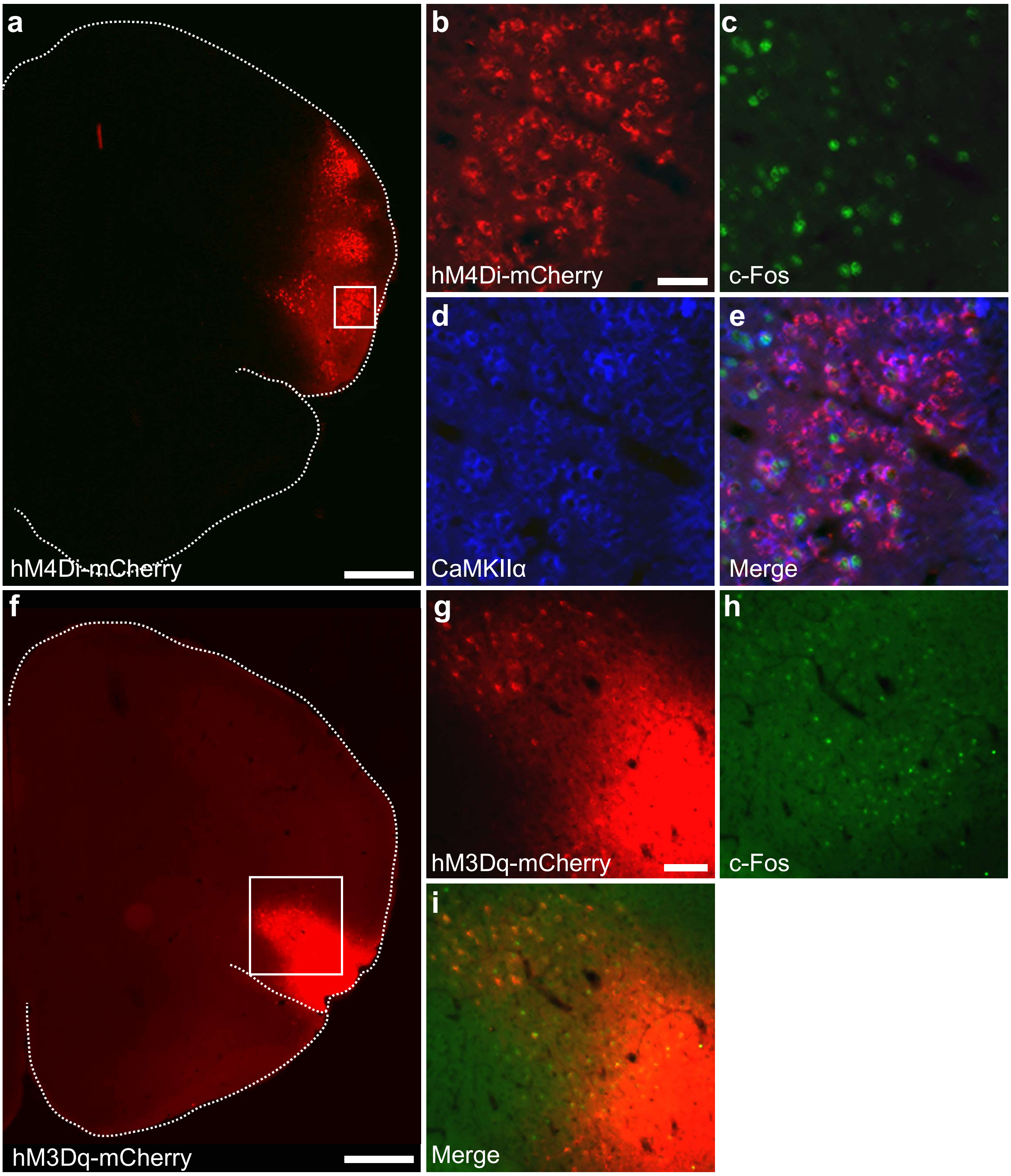

Fig. S2. Validation of DREADD manipulations efficiency. (**a**) Representative images of brain expressing hM4Di-mCherry. (**b–e**) Magnified immunostained images of the region highlighted by the white box in a, which show expression of the mCherry in red (b), c-Fos in green (c), CaMKIIα in blue (d), and a merge view of these markers (e). (**f**) Representative images of brain expressing hM3Dq-mCherry. (**g–i**) Magnified immunostained images of the region highlighted by the white box in f, which show expression of the mCherry in red (g), c-Fos in green (h), and a merge view of these markers (i). Scale bars, 500 µm (a and f), 50 µm (b), 100 µm (g).

Supplementary Fig. S3

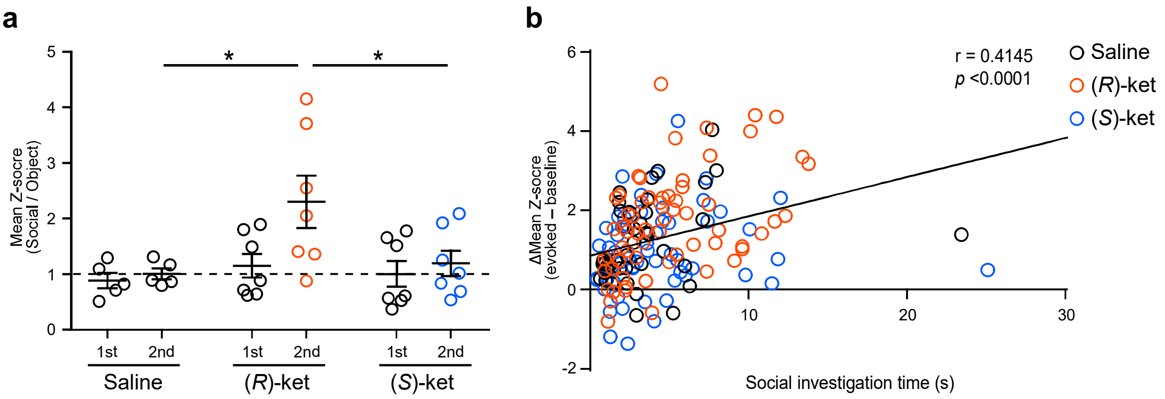

Fig. S3. aIC neuronal responses to social contact in social isolation-reared mice. (**a**) The neuronal activity ratio during contact with a mouse compared to that with an object in two three-chamber tests for social isolation-reared mice treated with the indicated drugs. Data are presented as the mean ± s.e.m. The data was calculated using the data from Fig. 3B to 3G. Statistical analysis was performed using Two-way repeated measured ANOVA, followed by Tukey´s multiple comparisons test: trial, F(1, 16) = 5.066, $p = 0.0388$; treatment, F(2, 16) = 3.782, $p = 0.0452$; interaction, F(2, 16) = 2.509, $p = 0.1128$. *$p < 0.05$. Data are presented as the mean ± s.e.m. (**b**) The graph shows the relationship between the duration for social investigation and the alteration in aIC activity during the 2nd three chamber test. This change in activity is calculated by subtracting the mean baseline z-score (from −3–0 seconds pre-contact) from the mean evoked z-score (from 0 to 3 seconds post-contact). The depicted line represents a linear regression of the combined data from all three groups which are mice treated with saline, (*R*)-ketamine, and (*S*)-ketamine. The statistical correlation was evaluated by Spearman correlation analysis.

Supplementary Fig. S4

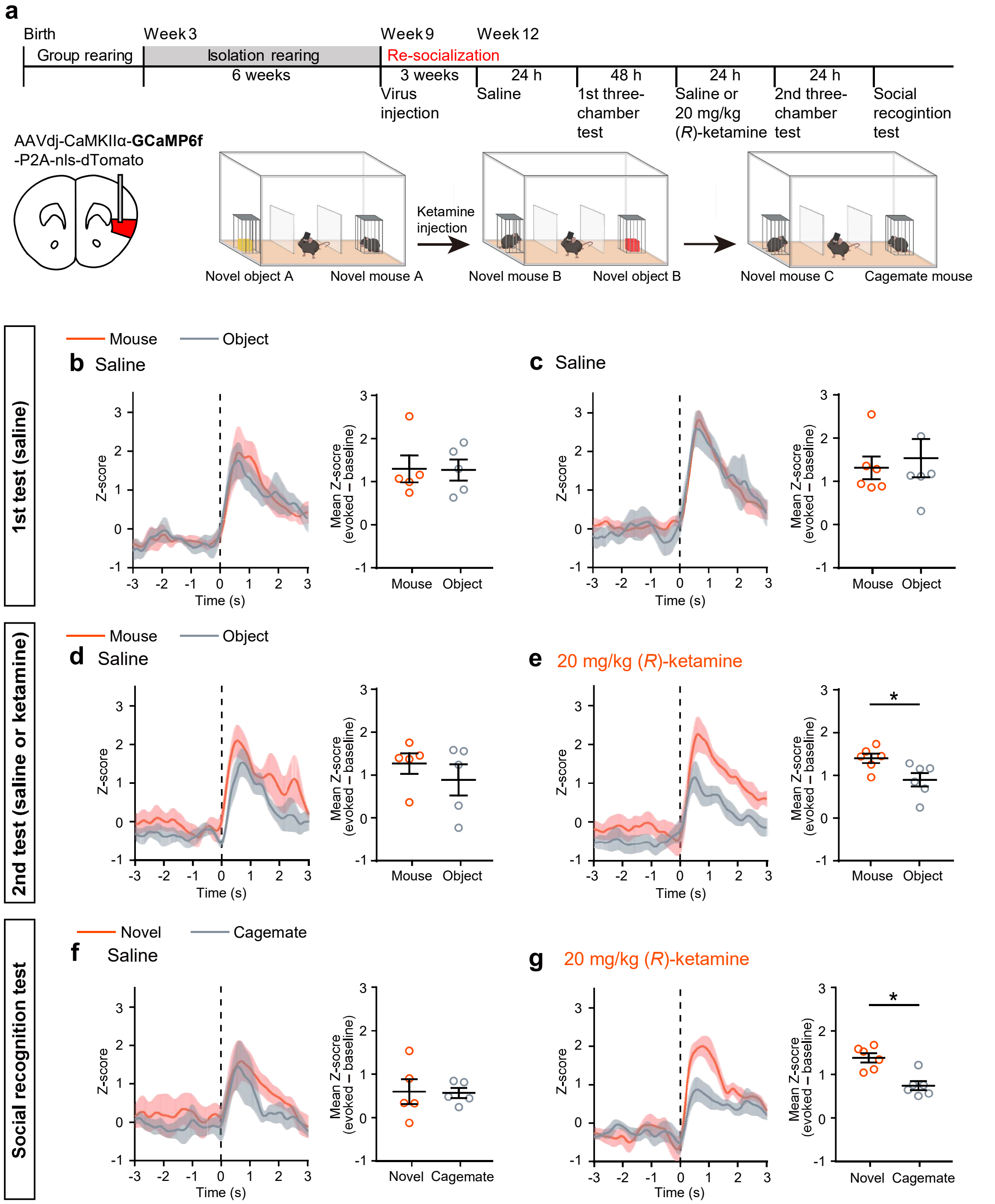

Fig. S4. (*R*)-ketamine enhances aIC neuronal responses during social investigation in re-socialized mice. (**a**) Experimental timeline and schematic of fiber photometry recording of the aIC activity in repeated three-chamber and social recognition tests with mice re-socialized after 6 weeks of social isolation rearing. (**b–e**) Neuronal activity of the aIC in nonsequential interaction with a novel mouse (orange) or object (gray) in the 1st test (b and c) and 2nd test (d and e) with saline (b and d, n = 5; c, n = 6) or 20 mg/kg (*R*)-ketamine (e) treatments. The right graphs show the quantification of social (orange) or object (gray) contact-evoked normalized GCaMP6f signals (mean evoked z-score of 0–3 seconds at post-contact subtracted by the mean baseline z-score of −3–0 seconds at pre-contact). Dashed lines indicate the onset of contact. Statistical analysis was performed using paired *t* test. *$p < 0.05$. (**f and g**) Neuronal activity of the aIC in nonsequential interaction with a novel (orange) or familiar (gray) mouse in the social recognition test with saline (f) or 20 mg/kg (*R*)-ketamine (g) treatments. The right graphs show the quantification of novel (orange) or familiar (gray) contact-evoked normalized GCaMP6f signals (mean evoked z-score of 0–3 seconds at post-contact subtracted by the mean baseline z-score of −3–0 seconds at pre-contact). Dashed lines indicate the onset of contact. Statistical analysis was performed using paired *t* test. *$p < 0.05$. Data are presented as the mean ± s.e.m.

Supplementary Fig. S5

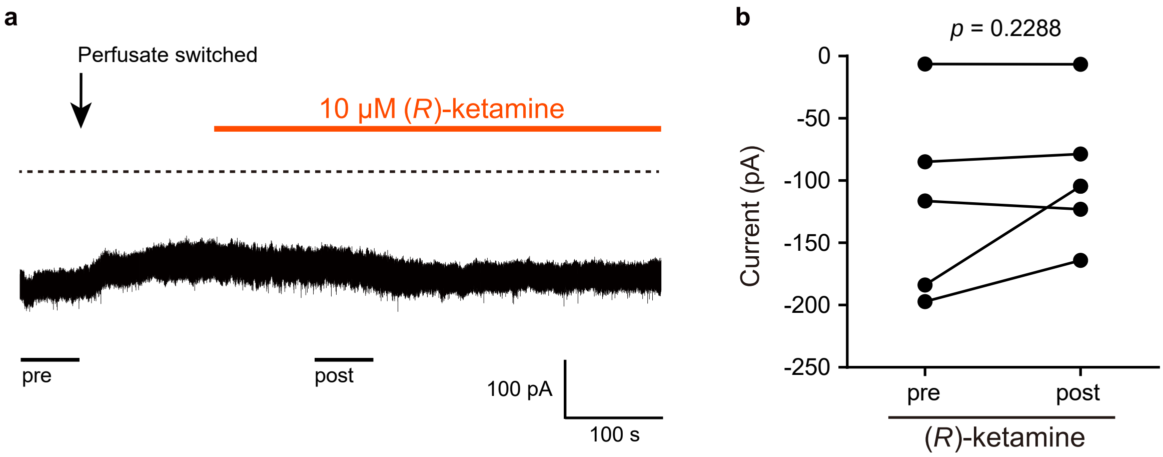

Fig. S5. Voltage-clamp recording from pyramidal neurons of the aIC. (**a**) A representative trace of (*R*)-ketamine application. The membrane potential is held at -80 mV in voltage-clamp recording and 10 μM (*R*)-ketamine-containing aCSF was applied after 1 min. (**b**) The graph shows the quantification of pre(basal current)- or post-(*R*)-ketamine treatment. Statistical analysis was performed using paired *t* test.

Supplementary Fig. S6

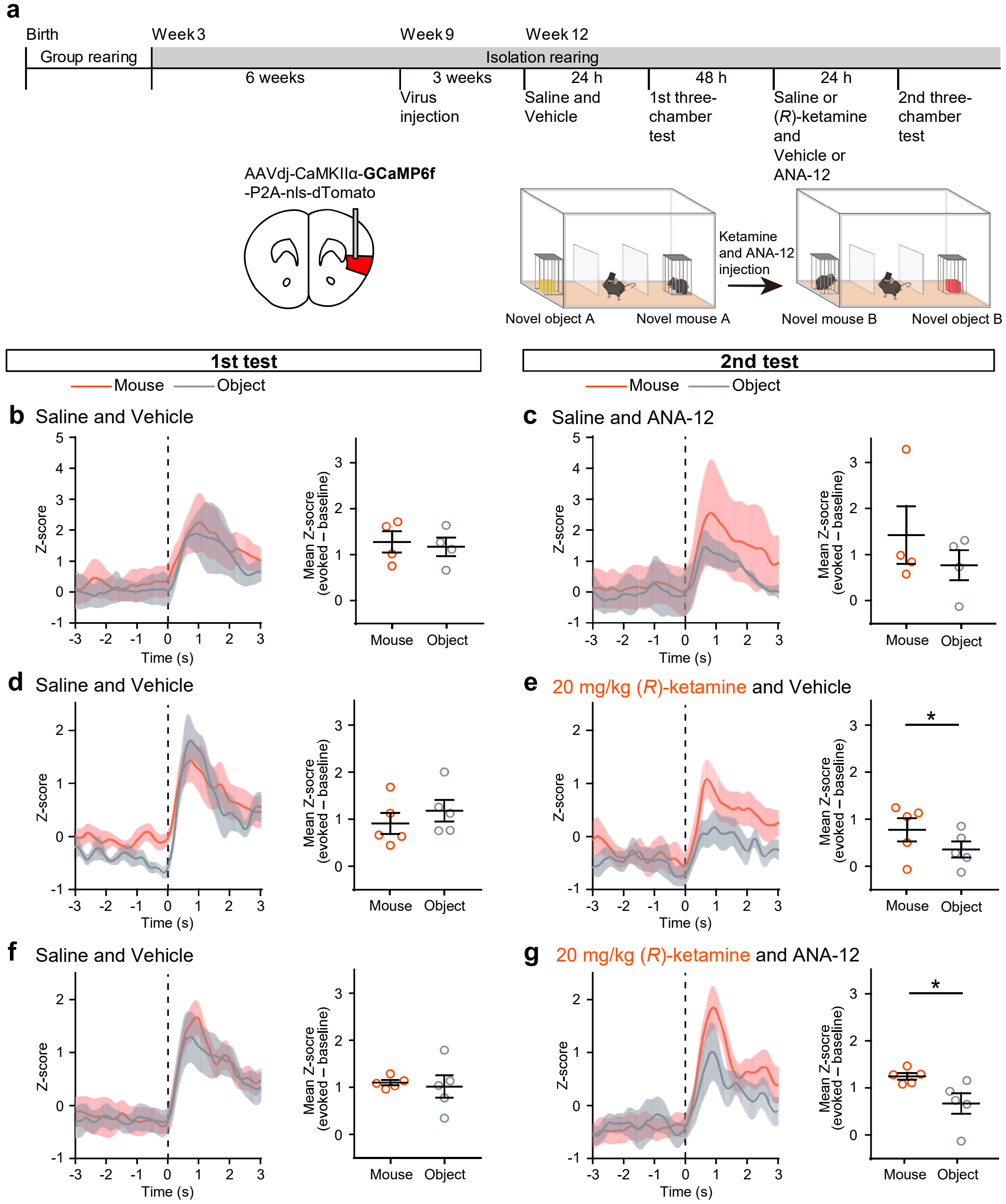

Fig. S6. No effect of TrkB inhibitor on the (*R*)-ketamine-induced restoration of aIC neuronal responses to the social investigation. (**a**) Experimental timeline of fiber photometry recording in social isolation-reared mice during the three-chamber test. (**b–g**) Neuronal activity of the aIC in social isolation-reared mice was observed during nonsequential interactions with a novel mouse or object in the 1st-three-chamber test with saline and vehicle treatment (b, d, and f) and the 2nd-three-chamber test with drug treatment. 0.5 mg/kg ANA-12 or vehicle was co-administered with saline or 20 mg/kg (*R*)-ketamine. (c, e, and g for saline and 0.5 mg/kg ANA-12, 20 mg/kg (*R*)-ketamine and vehicle, and 20 mg/kg (*R*)-ketamine and 0.5 mg/kg ANA-12; n = 4, 5, and 5, respectively). The right graphs show the quantification of social (orange) or object (gray) contact-evoked normalized GCaMP6f signals (mean evoked z-score of 0–3 seconds at post-contact subtracted by the mean baseline z-score of −3–0 seconds at pre-contact). Dashed lines indicate the onset of contact. Statistical analysis was performed using paired *t* test. *$p < 0.05$. Data are presented as the mean ± s.e.m.

Supplementary Fig. S7

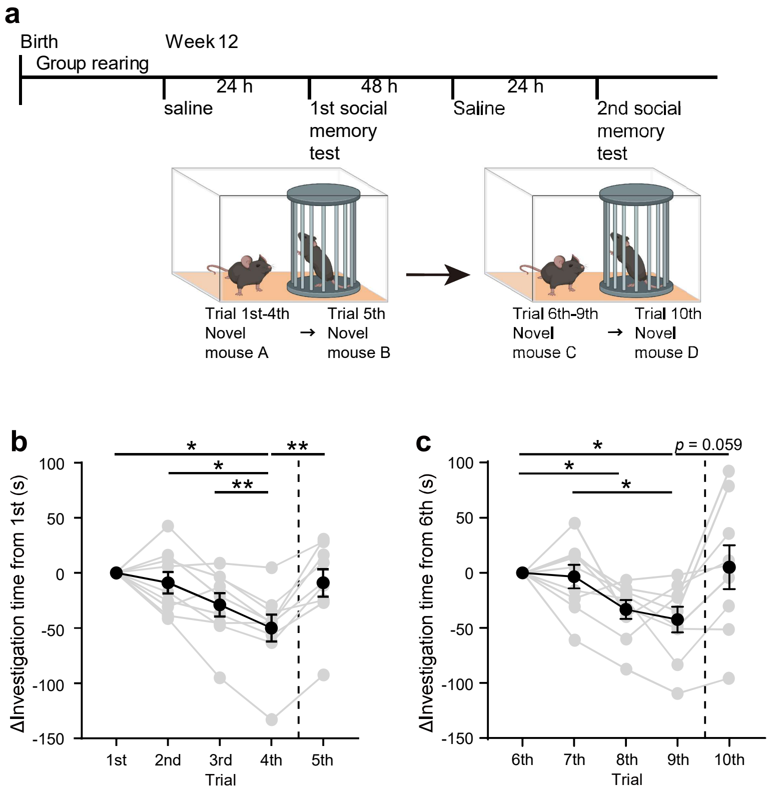

Fig. S7. Social memory tests in group-reared mice. (**a**) Experimental timeline and schematic of the 5-trial social memory test using group-reared mice. (**b and c**) Difference in investigation time between 1st trial and subsequent indicated trials during the 1st social memory test (b) and between 6th trial and indicated trials in the 2nd social memory test (c) 24 hours after saline treatments (n = 9). Statistical analysis was performed using one-way repeated measured ANOVA, followed by Tukey´s multiple comparisons test: trial, $F(2.419, 19.35) = 8.565$, $p = 0.0014$ (b); $F(2.179, 17.43) = 5.016$, $p = 0.0171$ (c). *$p < 0.05$, **$p < 0.01$. Data are presented as the mean ± s.e.m.

Supplementary Table S1. Abbreviations for brain areas

Abbreviation	Brain area
VIS	Visual cortex
LS	Lateral septal nucleus
DG	Dentate gyrus
HIP	Hippocampus
BLA	Basolateral amygdala nucleus
COA	Cortical amygdala
PTL	Parietal association cortex
RSP	Retrosplenial cortex
ENT	Entorhinal cortex
ECT	Ectorhinal cortex
CLA	Claustrum
CP	Caudoputamen
ACB	Nucleus accumbens
AUD	Auditory cortex
SS	Somatosensory cortex
ILA	Infralimbic cortex
PIR	Piriform cortex
ACC	Anterior cingulate cortex
IC	Insular cortex
ORB	Orbital cortex
PL	Prelimbic cortex
MO	Somatomotor cortex
